# Association between pain, physical performance, and kinesiophobia in older women with low back pain: A cross-sectional study

**DOI:** 10.1371/journal.pone.0337553

**Published:** 2026-02-27

**Authors:** Farzaneh Saki, Farzaneh Ramezani, Raziyeh Taheri

**Affiliations:** Department of Exercise Rehabilitation, Faculty of Sports Science, Bu-Ali Sina University, Hamedan, Iran; University of Tehran, IRAN, ISLAMIC REPUBLIC OF

## Abstract

**Background:**

Kinesiophobia is a psychological element that may contribute to persistent pain and functional decline in older women with chronic low back pain. Clarifying its role could inform more effective rehabilitation strategies.

**Objectives:**

To examine associations between kinesiophobia, pain intensity, and physical performance in older women with chronic low back pain.

**Methods:**

A cross-sectional study was conducted in 2024 with 119 women aged ≥ 60 years reporting nonspecific low back pain. Kinesiophobia was assessed using the Tampa Scale for Kinesiophobia (TSK-17), and pain with the Visual Analog Scale (VAS). The Timed Up and Go test and the 30-second sit-to-stand test were performed to evaluate balance and lower limb strength, respectively (physical performance). Correlation, regression, and mediation analyses were used to explore relationships among variables.

**Results:**

Higher pain intensity was strongly associated with higher kinesiophobia (β = 0.74, p = 0.001), while lower limb strength showed a negative association (β = −0.17, p = 0.005). No significant relationship emerged between kinesiophobia and balance (p > 0.05). Regression indicated that pain was the strongest predictor of kinesiophobia. Mediation analyses suggested that kinesiophobia fully explained the relationship between pain and reduced lower limb strength, while strength itself partially mediated the link between pain and kinesiophobia, highlighting both direct and indirect effects.

**Conclusions:**

In older women with chronic low back pain, kinesiophobia is positively related to pain severity and negatively related to lower limb strength, but not balance. Findings suggest fear of movement may mediate the impact of pain on physical performance, underscoring the importance of addressing psychological as well as physical factors in rehabilitation. Given the cross-sectional design, causal inferences should be drawn cautiously.

## Introduction

Kinesiophobia, defined as excessive, irrational fear of physical movement due to perceived risks of pain or re-injury [1], represents a major barrier to functional independence, particularly in older populations [2]. This fear typically develops following prolonged pain experiences, such as chronic low back pain, and manifests through movement avoidance and reduced physical activity [3–5]. Such behavioral patterns can profoundly compromise quality of life, limit participation in daily activities, and threaten functional autonomy in older individuals [6,7].

Low back pain affects up to 75% of older adults [8] and is frequently associated with decreased mobility, restricted social participation, and psychological sequelae, including anxiety, depression, and kinesiophobia [9–12]. Among individuals with persistent low back pain, kinesiophobia can intensify functional limitations and further reduce physical engagement by reinforcing avoidance behaviors [13,14]. Evidence indicates that kinesiophobia prevalence and severity may differ by sex, with women experiencing higher levels of fear-avoidance beliefs and associated disability compared to men [15,16]. A study reported that 80% of older women exhibited high kinesiophobia levels, which correlated with impaired physical function [3]. Despite this evidence, most existing research has examined heterogeneous populations or investigated psychological and physical factors separately, leaving the complex interplay between these domains inadequately understood, particularly among older women with chronic low back pain.

The relationship between kinesiophobia and physical function appears bidirectional and multifaceted. Research demonstrates that psychological factors such as kinesiophobia, pain catastrophizing, and anxiety can directly impair physical performance through avoidance behaviors and indirectly through deconditioning [17–19]. Conversely, poor physical performance, characterized by reduced balance, lower limb strength, and functional mobility, may reinforce fear beliefs, creating a self-perpetuating cycle of decline [20,21]. Physical performance is recognized as a key indicator of healthy aging [22], yet in individuals with persistent pain, performance is notably compromised [23]. Recent evidence suggests that interventions combining aerobic exercise with core stabilization can significantly improve physical performance and reduce fall risk in older adults with chronic nonspecific low back pain [24], highlighting the modifiable nature of these outcomes.

Performance-based assessments such as the Timed Up and Go (TUG) and 30-Second Sit-to-Stand (30s-STS) tests offer practical advantages for evaluating functional capacity in older adults [25,26]. These measures are simple, objective, require minimal equipment, and can be feasibly implemented across diverse clinical and care settings, including outpatient clinics, nursing homes, and home-based care [27,28]. Importantly, these tools capture functional dimensions, including balance, mobility, and lower limb strength, that are directly related to independence in activities of daily living [29–32]. However, the relative contribution of specific physical performance components (such as balance versus lower extremity strength) to kinesiophobia severity remains unclear [33]. Understanding which performance domains most strongly influence fear of movement could inform more targeted and efficient rehabilitation strategies.

Despite growing recognition of the biopsychosocial nature of chronic pain [34,35], research specifically examining the interconnections between pain intensity, multiple dimensions of physical performance, and kinesiophobia within older women with chronic low back pain remains limited. Previous studies have typically focused on younger or mixed-age populations [36,37], examined kinesiophobia in isolation from physical performance [38], or investigated pain and function without considering psychological mediators [39]. This represents a significant gap, as older women constitute a particularly vulnerable population: they experience higher rates of chronic low back pain, greater functional decline, and more severe psychosocial consequences compared to their male counterparts [15,40,41]. Furthermore, age-related physiological changes, including sarcopenia, reduced balance capacity, and altered pain processing, may uniquely influence the pain-kinesiophobia-function relationship in this demographic [42,43].

This study addresses these gaps by examining older women with chronic low back pain and exploring how pain intensity, physical performance domains, and demographic factors collectively relate to kinesiophobia within a comprehensive analytical framework. By utilizing accessible functional assessments applicable across diverse care settings and employing advanced statistical methods, this research demonstrates how modifiable physical factors may influence psychological outcomes. The findings have the potential to inform clinical practice by identifying which functional domains should be prioritized in rehabilitation programs to reduce fear-avoidant behaviors and improve outcomes in this vulnerable population.

Specifically, the study was designed to investigate the associations between pain intensity, physical performance, and kinesiophobia in older women with chronic low back pain, guided by the following hypotheses: (a) greater pain intensity would be associated with higher kinesiophobia levels, (b) poorer physical performance across multiple domains (balance, mobility, lower limb strength) would be associated with higher kinesiophobia levels, and (c) physical performance would partially mediate the relationship between pain intensity and kinesiophobia, such that higher pain would be associated with poorer performance, which in turn would be associated with greater kinesiophobia. By clarifying these interrelationships, the study aims to provide evidence for developing multidimensional rehabilitation strategies that concurrently address physical and psychological determinants of functional decline in older women with chronic low back pain.

## Materials and methods

### Study design

This cross-sectional study adhered to the STROBE guidelines for reporting observational research. Data were gathered from 119 older women at the Sports Rehabilitation Laboratory of Bu-Ali-Sina University between October and November 2024. The study complied with the Declaration of Helsinki and received approval from the Research Ethics Committee of Bu-Ali Sina University (IR.BASU.REC.1403.011). All participants provided written informed consent prior to their involvement.

### Participants

This study involved 119 older women with chronic nonspecific low back pain, recruited by referrals from physiotherapy clinics in Hamedan, based on predefined eligibility criteria. Each participant was clinically evaluated by a board-certified specialist in physical medicine and rehabilitation with expertise in musculoskeletal disorders. Chronic low back pain was diagnosed through a comprehensive clinical assessment, including detailed patient history and physical examination. Specific causes of low back pain, such as vertebral fractures, lumbar spinal stenosis, and disc herniation, were excluded based on clinical findings and, when necessary, review of recent imaging reports (such as X-ray, MRI). Inclusion criteria were: age over 60 years, low back pain lasting more than three months within the past year, at least two episodes of pain lasting two or more consecutive days, independence in daily activities, and no medical restrictions for physical activity. Exclusion criteria included diagnosed neurological disorders affecting mobility (for example, Parkinson’s disease, multiple sclerosis), vestibular disorders (for example, labyrinthitis, Meniere’s disease), cognitive impairment interfering with test participation, history of pelvic or spinal surgery, congenital spinal conditions (for example, scoliosis), falls within the past year, and any identifiable structural pathology. The specialist verifying eligibility was blinded to the study hypotheses and outcome measures to minimize bias. The same examiner conducted all evaluations.

### Measures

Demographic data, including age, height, weight, and BMI, were recorded. No participants were excluded based on their overweight or obesity status.

#### Dependent variable: Kinesiophobia.

The Tampa Scale for Kinesiophobia-17 (TSK-17) is a widely recognized and validated tool designed to evaluate fear of movement (kinesiophobia) in people with chronic low back pain. [44]. The scale comprises 17 items scored on a four-point Likert scale (from “strongly disagree” to “strongly agree”), assessing the degree to which individuals fear physical activity due to worries about potential injury or worsening pain. [45,46]. The questionnaire is designed to identify maladaptive beliefs and avoidance behaviors that may contribute to the persistence or chronicity of pain. Each item is rated on a 4-point scale, yielding a cumulative score between 17 and 68. Lower total scores reflect the absence or minimal presence of kinesiophobia, whereas higher scores represent more severe levels of kinesiophobic behavior [47]. A score above 37 indicates significant kinesiophobia [48]. In the present study, the validated Persian adaptation of the questionnaire was utilized. This version exhibited excellent internal consistency, as reflected by a Cronbach’s alpha coefficient of 0.94 [49].

#### Independent variables.

This study included three independent variables: 1) low back pain intensity, 2) dynamic balance, and 3) lower limb strength. Balance and strength were considered measures of physical performance*.*

#### Low back pain.

In this study, the intensity of low back pain was evaluated using the Visual Analog Scale (VAS), a validated tool consisting of a 10-centimeter horizontal line anchored by descriptors at each end: “no pain” (0) and “the most severe pain imaginable” (10). The participant indicates pain intensity by marking the line at the point that best represents her pain. This scale is highly sensitive to changes in pain and can be administered quickly without requiring complex equipment [50]. Previous studies have reported high reliability for this tool (test-retest correlation coefficient is usually higher than 0.80), and its validity has been confirmed by strong correlations with other tools, such as the Numerical Rating Scale (NRS) and the McGill Pain Questionnaire [51].

#### Physical performance measures.

*Timed Up and Go*

The Timed Up and Go (TUG) test was used to assess dynamic balance. Performance on this test may be influenced by factors beyond balance, such as pain intensity and kinesiophobia, which should be considered when interpreting associations with psychological outcomes. However, it may not detect subtle postural control deficits [52–54]. In this study, participants were instructed to stand up from a seated position in a standard armchair, walk a distance of 3 meters, turn around, return to the chair, and sit down again. Completion time was measured using a stopwatch [25]. Completion time exceeding 12 seconds among older adults indicates impaired balance and increased fall risk [55]. The TUG test has demonstrated high validity among older adults [25].

*30s-Sit-To-Stand*

The 30s-STS test was employed to evaluate functional lower limb strength, especially the quadriceps and gluteal muscles [28]. While this test reflects functional strength in daily activities, it does not isolate pure muscular strength and may be influenced by other factors such as pain intensity, kinesiophobia, and overall physical conditioning. These potential confounders were considered in the multivariable and mediation analyses, and the observed associations were interpreted with caution. Participants were seated in the center of an armless chair, ensuring their feet were flat on the floor and their arms were crossed over their chest. Within 30 seconds, participants completed as many full sit-to-stand repetitions as possible without using hand support. The total number of successful repetitions was considered a performance indicator, with higher number indicating better lower limb strength and functional ability [28].

### Procedures

After the volunteers attended the Sports Rehabilitation Laboratory of Bu-Ali Sina University, they received detailed information about the study’s procedures, objectives, and potential risks. All participants signed the written informed consent form. All assessments were performed in the controlled laboratory environment.

Initially, the demographic information of each participant was recorded. Then, participants completed the validated Persian version of the TSK-17 Questionnaire. Pain intensity was measured using the VAS. Participants then did two performance tests, the TUG and 30s-STS, in randomized order during the same session. To prevent the risk of injury, a 5-minute rest period was provided between each test. All assessments were performed by an experienced and trained therapist.

### Statistical analysis

The sample size was determined using G*Power software (version 3.1.9.2) for a multiple linear regression model with five predictors. Using the medium effect size of f² = 0.15 (the default medium effect size in G*Power for multiple regression), a significance level of 0.05, and 95% statistical power, a sample size of 119 participants was established. This sample size exceeds the minimum thresholds recommended by Green (1991), who suggests N ≥ 50 + 8m (where m is the number of predictors) for testing the overall model fit (50 + 8 × 5 = 90) and N ≥ 104 + m for testing individual regression coefficients (104 + 5 = 109), ensuring sufficient statistical power for reliable results [56].

Data analysis was conducted using SPSS software Version 26. The normality of residuals was assessed using the Shapiro-Wilk test and visual inspection of Q-Q plots and histograms. To examine the associations between variables, Spearman’s correlation test was applied. Multiple linear regression was employed to investigate the predictive effects of independent variables (pain intensity and physical performance) on the dependent variable (kinesiophobia). A p-value below 0.05 was deemed statistically significant.

To examine the direct and indirect relationships among pain intensity, physical performance, and kinesiophobia, mediation and moderation analyses were conducted using the PROCESS macro (Model 4 and Model 14). Bootstrapping with 5000 bias-corrected resamples was used to estimate the confidence intervals for indirect effects. Additionally, moderated mediation models were tested to explore conditional indirect effects based on demographic variables (age, BMI).

## Results

**Fig 1:**
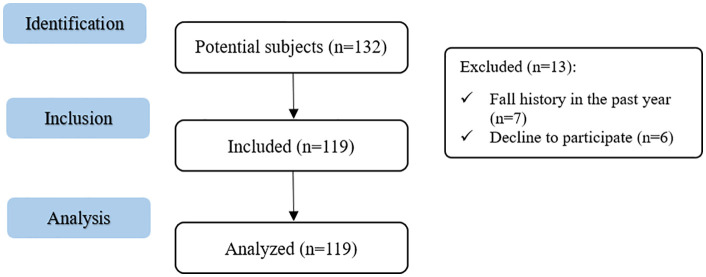
STROBE flow diagram for observational studies.

Out of 132 eligible older adults, 119 participants with chronic, nonspecific low back pain were included in the final sample (Fig 1).

### Demographic and clinical characteristics

Table 1 presents the demographic and clinical characteristics of the participants.

**Table 1:** Demographic and clinical characteristics of participants.

Variable	Sample (n = 119) Mean (SD)
Age (year)	67.42 (2.63)
Height (m)	1.62 (0.07)
Weight (kg)	72.71 (10.06)
BMI (kg/m2)	27.50 (3.81)
Pain intensity (VAS)	6.32 (1.56)
Balance (TUG (s))	10.13 (1.65)
Strength (30s-STS (repetitions))	7.18 (1.47)
Kinesiophobia (TSK-17)	51.90 (5.94)

BMI, Body Mass Index; VAS, Visual Analogue Scale; TUG, Timed Up and Go; 30s-STS, 30s Sit-To-Stand; TSK-17, Tampa Scale for Kinesiophobia-17 items.

### Bivariate association

Given the non-normal distribution of data, Spearman’s rank correlation was applied (Table 2). According to standard interpretation guidelines [57], correlations above 0.70 were considered strong, 0.40 to 0.69 moderate, and below 0.40 weak.

**Table 2:** Spearman’s correlation analysis between kinesiophobia, pain intensity, and physical performance (n= 119).

Variables	r	p-value	Strength of association
Pain intensity (VAS) – Kinesiophobia (TSK-17)	0.865	<0.001*	Strong positive
Strength (30s-STS) – Kinesiophobia (TSK-17)	−0.609	<0.001*	Moderate negative
Strength (30s-STS) – Pain intensity (VAS)	−0.558	<0.001*	Moderate negative
Balance (TUG) – Kinesiophobia (TSK-17)	0.030	0.745	Negligible (ns)
Balance (TUG) – Pain intensity (VAS)	−0.034	0.713	Negligible (ns)

* Correlation is significant at the 0.05 level.

ns, not significant.

VAS, Visual Analogue Scale; TSK-17, Tampa Scale for Kinesiophobia-17 items; 30s-STS, 30s Sit-To-Stand; TUG, Timed Up and Go.

**Fig 2:**
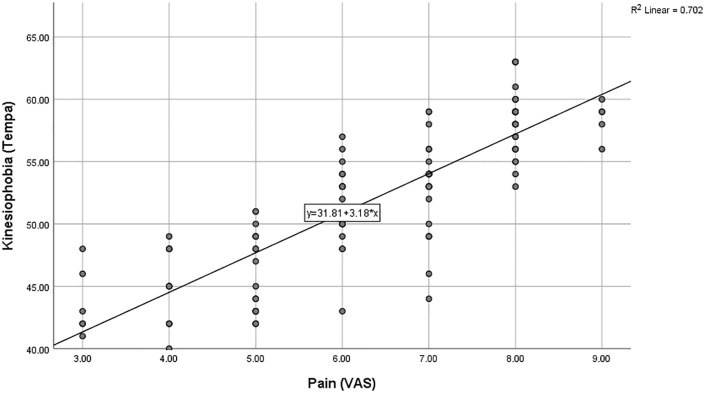
Shows a clear upward trend, confirming that higher pain intensity is associated with higher kinesiophobia.

The findings indicated a strong positive correlation between pain intensity and kinesiophobia (r = 0.86, p < 0.005), demonstrating that higher pain intensity was associated with increased kinesiophobia, as illustrated in Fig 2.

**Fig 3:**
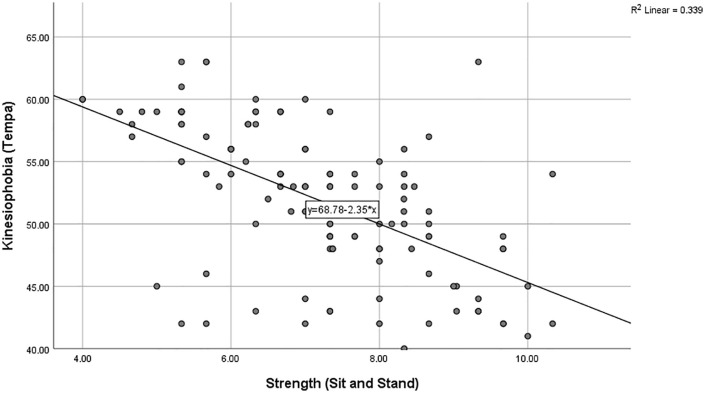
Shows a strong downward trend, suggesting that greater lower-limb strength is linked to lower kinesiophobia.

The findings revealed a moderate negative correlation between lower limb strength and kinesiophobia (r = −0.61, p < 0.005), suggesting that participants with higher lower limb strength exhibited reduced kinesiophobia, as illustrated in Fig 3.

Furthermore, a moderate negative correlation was found between lower limb strength and pain intensity (r = −0.56, p < 0.005), indicating that better lower limb strength is associated with reduced pain (Table 2). However, no significant correlations were found between balance and kinesiophobia (p > 0.05; Table 2).

### Multivariable linear regression analysis

To investigate the contribution of pain intensity, physical performance, and demographic factors to kinesiophobia, a multivariable linear regression analysis was performed. The model included pain intensity (VAS), lower limb strength (30-STS), balance (TUG), age, and BMI as independent variables. All model assumptions were met: residuals were normally distributed, no influential outliers were detected, and multicolinearity was ruled out (all VIF values < 2).

The regression model significantly predicted kinesiophobia scores (F (5, 113) = 60.01, p < 0.001), accounting for 72.6% of the variance (R² = 0.726). Among the predictors, pain intensity showed a strong and positive association with kinesiophobia (β = 0.74, p < 0.001), indicating that higher pain levels were linked to greater fear of movement (Table 3). Lower limb strength was negatively and significantly associated with kinesiophobia (β = −0.17, p = 0.005), suggesting that reduced lower limb strength may contribute to higher fear of movement (Table 3). In contrast, balance performance (TUG) did not appear as a significant predictor (p = 0.212; Table 3). Age and BMI were included to control for potential demographic confounders; however, they were not significantly associated with kinesiophobia (p > 0.05; Table 3).

**Table 3:** Association of pain, physical performance, and demographic factors with kinesiophobia in multivariable linear regression analysis (n = 119).

Predictor	B	SE B	Beta	t	p	VIF
(Constant)	32.76	8.96	–	3.65	0.001*	–
Age (year)	0.07	0.11	0.03	0.60	0.556	1.04
BMI (kg/m2)	−0.02	0.07	−0.02	−0.31	0.756	1.03
Pain intensity (VAS)	2.82	0.22	0.74	12.51	0.001*	1.46
Balance (TUG (s))	0.22	0.17	0.06	1.25	0.212	1
Strength (30s-STS (repetitions))	−0.68	0.24	−0.17	−2.85	0.005*	1.46

VAS, Visual Analogue Scale; TUG, Timed Up and Go; 30s-STS, 30s Sit-To-Stand.

### Mediation analysis

Two mediation models were tested to examine the indirect effects of pain on physical performance through kinesiophobia and lower limb strength (Table 4).

**Table 4:** Mediation models testing indirect effects.

Model	Path	Effect of predictor on mediator (a)	Effect of mediator on outcome (b)	Direct effect of predictor on outcome (c′)	Indirect Effect (a × b)	BootCI 95%	Total effect (c′ + (a × b))	Mediation type
1	Pain → Kinesiophobia → Sit-to-Stand	3.18	−0.09	−0.22	−0.30	[-0.56, -0.08]	−0.52	Full
2	Pain → Sit-to-Stand → Kinesiophobia	−0.52	−0.66	2.82	+0.35	[0.07, 0.74]	3.17	Partial

**Fig 4:**
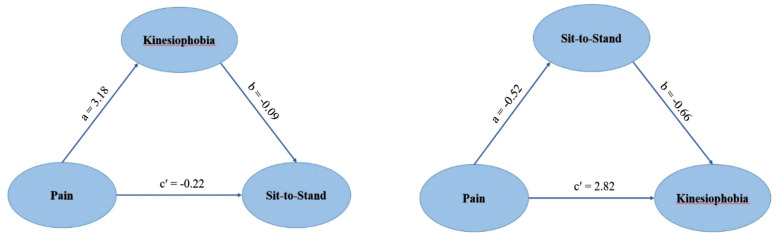
Indirect effects of pain on physical performance via kinesiophobia and lower limb strength. (a) Path diagram of Mediation model 1 (b) Path diagram of Mediation model 2.

In the first model, kinesiophobia (fear of movement) was entered as a mediator between pain and STS (lower limb strength). The indirect effect was statistically significant (β = −0.30, BootCI [−0.56, −0.08]), while the direct effect was non-significant (β = −0.22, p = .08). This suggests that pain may influence lower limb strength through impact on fear of movement (Fig 4a).

In the second model, STS (lower limb strength) was tested as a mediator between pain and kinesiophobia. Both the direct (β = 2.82, p < .001) and indirect effects (β = 0.35, BootCI [0.07, 0.74]) were statistically significant, indicating partial mediation. These findings suggest that pain affects kinesiophobia both directly and indirectly through lower limb performance (Fig 4b).

Bootstrapping with 5000 bias-corrected resamples was used to estimate 95% confidence intervals (CI) for the indirect effects. Additional analyses were conducted to test moderation and moderated mediation effects (such as age, BMI), but none reached statistical significance. No missing data were present in the variables included in the mediation and moderation analyses.

## Discussion

The aim of this study was to investigate the associations between pain intensity, physical performance, and kinesiophobia in older women with chronic nonspecific low back pain. Our findings demonstrated that pain intensity was the strongest predictor of kinesiophobia, and lower limb strength (as measured by 30s-STS) was inversely related to kinesiophobia. In contrast, balance (as measured by TUG) was not significantly associated with kinesiophobia. Moreover, mediation analyses revealed that kinesiophobia fully mediated the relationship between pain and lower limb strength, while lower limb strength partially mediated the relationship between pain and kinesiophobia. These findings emphasize both direct and indirect pathways through which pain contributes to performance limitations in this population.

The strong association between pain intensity and kinesiophobia aligns with the fear-avoidance model, which posits that pain triggers maladaptive beliefs and catastrophic thoughts, leading to avoidance behaviors, reduced activity, and progressive deconditioning [13,58,59]. This finding is consistent with previous research demonstrating that individuals with chronic low back pain (CLBP) frequently associate physical movement with pain exacerbation, creating a self-reinforcing cycle of fear and disability [4,5]. The magnitude of this association underscores its substantial influence on fear-related cognitions. Neurobiological evidence provides additional support for this relationship. Pain and fear share common neural substrates, including the amygdala, anterior cingulate cortex, and prefrontal regions, which are involved in threat processing and emotional regulation [60–62]. Functional neuroimaging studies have shown that individuals with high kinesiophobia exhibit heightened activation in these regions when anticipating movement, suggesting that fear of pain may be neurologically reinforced through repeated pain experiences [63,64]. In older adults, age-related changes in central pain processing, including altered descending inhibition and increased central sensitization, may further amplify the pain-fear relationship [65]. It is important to consider potential bidirectional influences. While our cross-sectional design does not permit causal inference, longitudinal research suggests that kinesiophobia can also amplify pain perception through attentional bias and hypervigilance to bodily sensations [66,67]. This reciprocal relationship may be particularly pronounced in older women, who demonstrate higher pain sensitivity and greater emotional reactivity to pain compared to men and younger populations [68,69].

Lower limb strength demonstrated a significant but modest inverse relationship with kinesiophobia. While statistically significant, this effect size is considerably smaller than that of pain intensity, suggesting that functional strength plays a secondary role in determining fear of movement. The moderate effect size may reflect several underlying mechanisms. First, the 30s-STS test, although functional and clinically practical, does not isolate pure muscular strength and may be influenced by pain tolerance, motivation, and movement strategy [70,71]. Individuals with high kinesiophobia may perform the test cautiously, limiting repetitions not due to actual strength deficits but due to fear-driven protective behaviors [72]. Second, our sample may have exhibited sufficient functional strength for basic mobility tasks, resulting in a restricted range that attenuated correlations with kinesiophobia [73]. Previous research supports the link between kinesiophobia and impaired muscle function in CLBP populations [4,5,33]. Severeijns et al. (2005) found that fear-avoidance beliefs predicted greater functional disability independent of pain intensity, highlighting the role of psychological factors in performance decrements [17]. The findings of Alshehri et al. (2024) also showed that kinesiophobia directly and indirectly affects proprioception and balance through pain intensity, functional mobility, and mental health status [74]. Our mediation analysis extends this work by demonstrating that pain may reduce functional strength primarily through its influence on fear of movement. Specifically, higher pain intensity was associated with greater kinesiophobia, which in turn predicted lower strength, suggesting that interventions targeting kinesiophobia may interrupt this pathway and preserve physical function [36,75].

Unexpectedly, no significant relationship was found between balance (TUG performance) and kinesiophobia. This contrasts with studies reporting that fear of movement influences proprioception and postural control in musculoskeletal pain populations [76]. Several methodological and population-specific factors may explain this null finding. The TUG test, while widely used in this population, may lack the sensitivity required to detect subtle postural control deficits in relatively high-functioning older adults [77,78]. Our participants demonstrated preserved mobility (mean TUG = 10 s), almost near to clinical cutoff for fall risk (>12 seconds) [55]. This suggests our sample maintained adequate dynamic balance for basic mobility tasks, resulting in restricted variance that may have obscured associations with kinesiophobia. Studies employing more sensitive balance assessments, including static single-leg stance, tandem balance, instrumented posturography, and force plate analysis, have successfully identified balance-kinesiophobia relationships even in functionally preserved populations [79]. Furthermore, the TUG primarily assesses dynamic mobility rather than isolated postural stability [80]. It incorporates multiple functional components (sit-to-stand transition, gait, turning, spatial orientation) that may dilute specific balance deficits [81]. In contrast, static balance measures and tests requiring greater postural challenge (for example, standing on foam, eyes closed conditions) may better capture fear-related postural adaptations [82].

Our findings align with some previous research. Karos et al. (2017) reported no significant correlation between kinesiophobia and basic balance measures in community-dwelling older adults with musculoskeletal pain, suggesting that the pain-fear-balance relationship may be task-specific or population-dependent [83]. Conversely, studies demonstrating balance-kinesiophobia associations often involved populations with more severe impairments or employed different assessment methods. Alshehri et al. (2024) found that kinesiophobia indirectly affected balance through its impact on proprioception and mental health in post-surgical hip replacement patients, a population with more pronounced postural challenges than our community-dwelling sample [74]. Ishak et al. (2017) reported that kinesiophobia predicted diminished mobility in broader CLBP cohorts but used self-reported balance confidence rather than objective performance measures [84].

It is also possible that older women with CLBP develop compensatory movement strategies that maintain balance performance despite elevated kinesiophobia [85,86]. Research indicates that individuals with chronic pain often adopt cautious movement patterns characterized by slower gait, wider base of support, and reduced center of mass displacement, strategies that preserve stability but compromise movement efficiency [87,88]. Such compensations might maintain TUG performance within normal ranges while kinesiophobia remains elevated, obscuring the expected relationship.

Our mediation analyses revealed bidirectional pathways that provide novel insights into the mechanisms linking pain, kinesiophobia, and physical performance. In the primary mediation model, kinesiophobia fully mediated the relationship between pain intensity and lower limb strength. This suggests that pain’s impact on functional strength operates almost entirely through fear of movement rather than through direct biomechanical or physiological impairments.

In the reverse mediation model, lower limb strength partially mediated the pain-kinesiophobia relationship. This indicates that pain increases kinesiophobia both directly (through threat appraisal and fear conditioning) and indirectly (through pain-induced strength deficits that reinforce perceptions of vulnerability).

The bidirectionality observed in our analyses suggests a self-perpetuating cycle: pain increases kinesiophobia, which promotes avoidance and reduces strength, and reduced strength further reinforces kinesiophobia by confirming fears of physical vulnerability. This vicious cycle aligns with the fear-avoidance model and highlights multiple potential intervention points [14,20,89]. Breaking this cycle may require simultaneous targeting of pain, fear-related cognitions, and physical performance deficits rather than addressing each component in isolation.

The findings of this study highlight pain intensity as the primary predictor of kinesiophobia in older women with low back pain, yet they also suggest that enhancing lower limb strength may offer additional benefits in reducing fear of movement. Therefore, effective interventions should adopt a comprehensive approach that prioritizes pain management while incorporating progressive strengthening exercises, pain education, and psychological strategies aimed at reducing kinesiophobia. Given its mediating role, addressing kinesiophobia as a core component of rehabilitation may improve both psychological resilience and physical performance in this population.

### Strengths and limitations

A key strength of this study lies in the use of standardized, simple, and clinically applicable measures (VAS, TUG, 30s-STS, and TSK-17), which can be performed in different clinical and community settings. Another strength is the inclusion of mediation models, which provide novel insights into the mechanisms linking pain, physical performance, and kinesiophobia in older women.

Several limitations should be acknowledged. First, the cross-sectional design of this study limits causal inference; longitudinal research is needed to establish temporal relationships and confirm directional pathways between variables. Second, potential selection bias may have occurred due to the recruitment of participants from a single geographic region and through physiotherapy clinics, which may limit the representativeness of the sample. Third, although the study focused on physical factors, it did not control for other psychological variables such as depression, anxiety, or pain catastrophizing, which may confound the observed associations. Fourth, reliance on self-reported measures (TSK-17, VAS) may introduce recall and reporting biases. Fifth, while the TUG test is widely used, it may lack the sensitivity to detect subtle postural control deficits in this cohort. Similarly, the 30s-STS test, although practical and functional, does not isolate pure muscular strength and may be influenced by pain and psychological factors. Future studies are encouraged to incorporate more precise strength assessments (e.g., isokinetic dynamometry), control for psychological variables, physical activity level, and adopt longitudinal designs with more diverse populations to enhance external validity and deepen understanding of biopsychosocial interactions.

## Conclusions

In summary, pain intensity and lower limb strength were significantly associated with kinesiophobia in older women with chronic low back pain, whereas balance showed no significant relationship. Mediation analyses further revealed that fear of movement plays a central role in explaining how pain impacts physical performance. These findings underscore the importance of incorporating psychological components, such as fear reduction strategies, into rehabilitation programs alongside traditional physical strengthening approaches. Clinicians should adopt a comprehensive, multidimensional framework that targets both physical and psychological contributors to disability in this population.

*Patient consent for publication:* Consent obtained directly from patient(s).

*Provenance and peer review:* Not commissioned; externally peer reviewed.

*Patient and public involvement:* Patients and/or the public were not involved in the design, conduct, reporting, or dissemination plans of this research.
